# Synthesis of Polymer Precursor 12-Oxododecenoic Acid Utilizing Recombinant Papaya Hydroperoxide Lyase in an Enzyme Cascade

**DOI:** 10.1007/s12010-022-04095-0

**Published:** 2022-07-29

**Authors:** Anna Coenen, Valentin Gala Marti, Kira Müller, Maria Sheremetiev, Lorenzo Finamore, Ulrich Schörken

**Affiliations:** TH Köln - Campus Leverkusen, Campusplatz 1, 51379 Leverkusen, Germany

**Keywords:** Hydroperoxide lyase, 12-Oxododecenoic acid, Hexanal, Enzyme cascade, Polymer precursor

## Abstract

Hydroperoxide lyases (HPLs) catalyze the splitting of 13S-hydroperoxyoctadecadienoic acid (13S-HPODE) into the green note flavor hexanal and 12-oxo-9(Z)-dodecenoic acid, which is not yet used industrially. Here, HPL from *Carica papaya* (HPL_CP_) was cloned and functionally expressed in *Escherichia coli* to investigate synthesis of 12-oxo-9(Z)-dodecenoic acid in detail. To improve the low catalytic activity of full-length HPL_CP_, the hydrophobic, non-conserved N-terminal sequence was deleted. This enhanced enzyme activity from initial 10 to 40 U/l. With optimization of solubilization buffer, expression media enzyme activity was increased to 2700 U/l. The tetrameric enzyme was produced in a 1.5 l fermenter and enriched by affinity chromatography. The enzyme preparation possesses a slightly acidic pH optimum and a catalytic efficiency (k_cat_/K_M_) of 2.73 × 10^6^ s^−1^·M^−1^ towards 13S-HPODE. Interestingly, HPL_CP-N_ could be applied for the synthesis of 12-oxo-9(Z)-dodecenoic acid, and 1 mM of 13S-HPODE was transformed in just 10 s with a yield of 90%. At protein concentrations of 10 mg/ml, the slow formation of the 10(E)-isomer traumatin was observed, pointing to a non-enzymatic isomerization process. Bearing this in mind, a one-pot enzyme cascade starting from safflower oil was developed with consecutive addition of *Pseudomonas fluorescens* lipase, *Glycine max* lipoxygenase (LOX-1), and HPL_CP-N_. A yield of 43% was obtained upon fast extraction of the reaction mixtures after 1 min of HPL_CP-N_ reaction. This work provides first insights into an enzyme cascade synthesis of 12-oxo-9(Z)-dodecenoic acid, which may serve as a bifunctional precursor for bio-based polymer synthesis.

**Supplementary Information:**

The online version contains supplementary material available at 10.1007/s12010-022-04095-0.

## Introduction

Polymer production is largely dependent on petrochemical raw materials, and the share of bio-based polymers is still below 2% [1]. Climate change and depletion of fossil fuels urge a major shift in the chemical industry towards renewable carbon-based products. In this respect, vegetable oils are well suited for the synthesis of biogenic specialty polymers. Bifunctional intermediates including dicarboxylic acids or ω-hydroxy acids were synthesized successfully in reaction cascades utilizing the Baeyer–Villiger monooxygenase (BVMO) catalyzed oxidative rearrangement reactions [2–4] or the ω-terminal oxidation of fatty acids [5, 6]. The consecutive transformation of 12-hydroxystearic acid with alcohol dehydrogenase (ADH), BVMO, and lipase leads to 11-hydroxyundecanoic acid. This intermediate was then turned into 11-aminoundecanoic acid with a genetically engineered *E. coli* expressing ADH and transaminase [7]. The reaction cascade proceeded to 11-oxoundecanoic acid via oxidation followed by transaminase catalyzed amination. The synthesis of bio-based 12-aminododecanoic acid (12-aminolauric acid) was enabled with an engineered whole-cell biocatalyst by combining ω-oxidation of lauric acid to 12-oxododecanoic acid with subsequent transaminase catalysis [8–10]. 12-Aminolauric acid is a suitable intermediate for synthesis of polyamide Nylon 12. However, lauric acid has only limited availability from tropical coconut and palm kernel fruits. Therefore, alternative biogenic raw materials, which do not threat pristine rainforest areas, are needed for the synthesis of bifunctional C12-intermediates for, e.g., Nylon synthesis.

Recently, the combination of lipase and lipoxygenase (LOX) with green surfactant and in situ oxygen generation was developed by our group for high-yield synthesis of 13S-HPODE from safflower oil [11]. The subsequent HPL cleavage of 13S-HPODE leads to 12-oxododecenoic acid and hexanal. While green note aromas such as hexanal and hexenal are already utilized in flavor and food industry, only little attention is paid to the synthesis of 12-oxododecenoic acid [12–14]. Thus, only a third of the linoleic acid starting material is exploited so far. We suggest that the second reaction product 12-oxododecenoic acid is applied for polymer synthesis. Well-known reduction, oxidation, or transamination processes lead to useful intermediates like 12-hydroxydodecanoic acid, dodecanedioic acid, or ω-aminododecanoic acid for Nylon-12 production. Similarly, 9-oxononanoic acid was synthesized in a coupled enzymatic reaction with 9-LOX and 9/13-HPL and was proposed as precursor for biopolymers [15].

HPLs are heme- and iron-binding proteins of the cytochrome P450 family and belong to the subclass CYP74 [16, 17]. They catalyze the formation of short-lived fatty acid hemiacetals from their hydroperoxide substrates, which are further cleaved into aldehydes and ω-oxoacids [18]. Depending on their sequence homologies, HPLs are divided into the subclasses CYP74B (13-HPLs) and CYP74C (9-HPLs and mixed 9/13-HPLS), which catalyze the cleavage of 9- or 13-hydroperoxides either to C9-aldehydes and C9-oxoacids or C6-aldehydes and C12-oxoacids [19–22]. HPLs were identified in various plants such as guava, tomato, alfalfa, or cucumber. They have been extracted from plant tissue or expressed recombinantly and purified for further characterization [23–26]. The aldehyde reaction products and their derivatives are called green leaf volatiles (GLVs) and play an important role in plant defense against pathogen and herbivore attacks [27–29]. In recent years, interest in HPLs was driven by the synthesis of GLVs as valuable products for the food and flavor industry exhibiting fresh green to cucumber-like scents. Especially HPL from guava is utilized industrially for green note production. This enzyme has either been extracted from fruit tissue or recombinantly expressed in *E. coli* [23, 30]. A genetically optimized HPL from guava was expressed with improved stability [31]. Little attention was paid so far to the co-product 12-oxododecenoic acid, which is naturally isomerized to traumatin, an important phytohormone for wound healing [32].

**Fig. 1:**
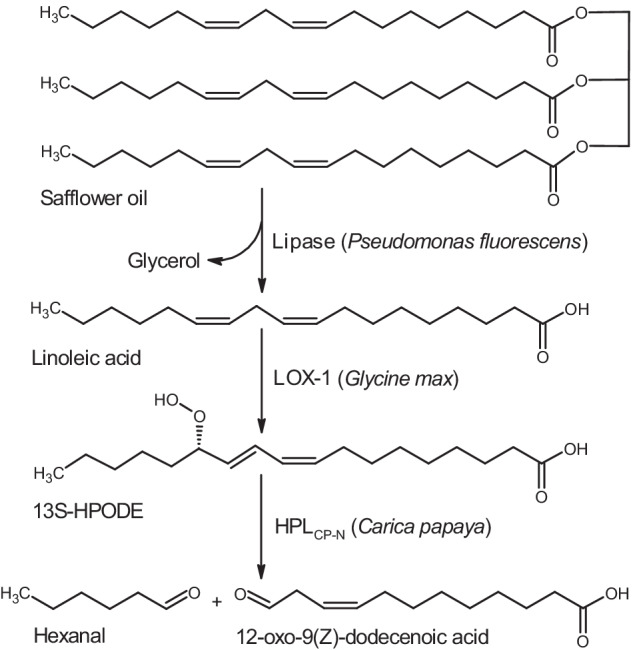
Enzyme cascade for the production of 12-oxo-9(Z)-dodecenoic acid starting from safflower oil (exemplified as trilinolein) utilizing lipase from *P. fluorescens*, lipoxygenase 1 from soybean, and N-terminally truncated hydroperoxide lyase from papaya

Here we present the cloning, expression, and characterization of papaya HPL as well as evaluation of its 12-oxododecenoic acid synthesis starting from 13S-hydroperoxyoctadecadienoic acid (13S-HPODE). The objective of the work was the synthesis of a bifunctional C12-intermediate suitable for polymer application without using tropical lauric acid–rich oils. For this, we developed an enzyme cascade with N-terminally truncated papaya HPL, soybean LOX-1, and *P.* *fluorescens* lipase for multi-step transformation starting from safflower oil (Fig. 1).

## Materials and Methods

### Reagents

Safflower oil was purchased from Gefro (Germany) and had a fatty acid composition of 77.2% linoleic acid, 13.3% oleic acid, 2.4% stearic acid, 6.7% palmitic acid, and 0.4% of other fatty acids [11]. 13S-HPODE, 12-oxo-9(Z)-dodecenoic acid, and 12-oxo-10(E)-dodecenoic acid standards were from Larodan (Sweden). Hexanal, hexanol, and 12-hydroxydodecanoic acid standards, *Glycine max* LOX-1, and *P. fluorescens* Amano lipase were obtained from Sigma-Aldrich (USA). Linoleic acid was purchased from Thermo Fisher Scientific (USA), and δ-aminolevulinic acid (ALA), Triton X-100, isopropyl β-d-1-thiogalactopyranoside (IPTG), as well as kanamycin sulfate and ampicillin sodium salt were obtained from Carl Roth (Germany). All other solvents and chemicals were supplied by Carl Roth (Germany), Sigma-Aldrich (USA), or Thermo Fisher Scientific (USA). For PCR, Phusion Hot Start DNA polymerase with 5 × Phusion High-fidelity buffer and dNTPs were obtained from Thermo Fisher Scientific (USA). PageRuler™ Prestained Protein ladder, restriction enzymes, and the monoclonal AP-conjugated Anti-His (C-term) antibody (AB_2556555) were purchased from Thermo Fisher Scientific (USA) as well. Alkaline phosphatase was obtained from New England Biolabs GmbH (Germany).

### Bioinformatic Analyses

*Carica papaya* HPL sequence (accession number: XP_021890218.1) was identified through BLAST analysis with the “National Center for Biotechnology Information” (NCBI) website by searching for putative HPL sequences of the already analyzed guava HPL (accession number: AAK15070.1). A multiple sequence alignment of protein sequences was performed with ClustalW [33] using the BLOSUM62 matrix. A phylogenetic tree was created with ClustalX [34] and NJPlot [35] with the neighbor-joining algorithm. The bootstrap value was set to 1000.

### Cloning and Expression of HPL

All strains, vectors, and oligonucleotides are listed in Table 1. Oligonucleotides were synthesized by Eurofins genomics (Germany). The *hpl*_CP_ gene was codon-optimized for expression in *E. coli* and synthesized with a C-terminal hexahistidine tag through gene synthesis by BioCat GmbH (Germany). The gene was cloned into the expression vector pET-28a( +) (Fig. S1). The non-conserved, hydrophobic N-terminus was identified with a multiple sequence alignment and deleted (Fig. S1, Fig. S2) by PCR with oligonucleotides binding at the end of the N-terminus. The Phusion Hot Start DNA polymerase with 5 × Phusion High-fidelity buffer and dNTPs (Thermo Fisher Scientific, USA) was used in the PCR. The truncated fragment was ligated into the cloning vector pJET1.2/blunt (Thermo Fisher Scientific, USA), and *E. coli* XL1-Blue was transformed with the respective vector and cultivated overnight at 37 °C in LB + 100 µg/ml ampicillin. The vector was extracted and restricted with *Bam*HI and *Nde*I. The *hpl*_CP-N_ gene was then ligated into the expression vector pET-28a( +). *E. coli* BL21(DE3) [36] was transformed with the full-length as well as the truncated *hpl* vectors.

**Table 1:**
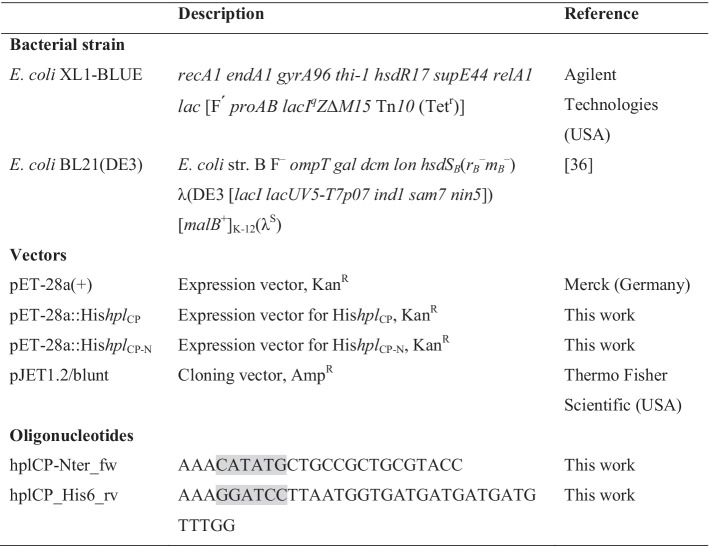
Strains, vectors, and oligonucleotides used in the experiments. Restriction sites are highlighted in gray. *Kan*^*R*^ kanamycin resistance, *Amp*^*R*^ ampicillin resistance

Cells expressing HPL were cultivated in 500-ml shaking flasks in 50-ml lysogeny broth (LB), terrific broth (TB), or ZYM5052 (compositions of cultivation media are listed in Table S1) with 50 µg/ml kanamycin. Optionally, 2.5 mM δ-aminolevulinic acid and 0.1 mM ammonium ferric citrate were added. The shaking flasks were inoculated with 2% (v/v) bacteria from an overnight culture. Cells were cultivated at 37 °C and 200 rpm until OD_600_ of 0.6 (for cultivation in LB) or 1 (for cultivation in TB and ZYM5052) was reached. Protein expression in LB and TB broth was induced by addition of 1 mM isopropyl β-d-1-thiogalactopyranoside (IPTG), and temperature was decreased to 25 °C. Cells were cultivated for 24 h. Then, cells were harvested by centrifugation at 4500 × *g* for 15 min and frozen at − 20 °C until further use. Cell pellets were suspended in 10 ml buffer (50 mM potassium phosphate buffer pH 6 with 1 M NaCl and 0.2% Triton X-100 if not noted otherwise) and disrupted by ultrasound sonication for 105 s in seven intervals of 15 s. The soluble fraction (SF) was obtained from the crude extract (CE) by centrifugation for 45 min with 21,000 × *g* at 4 °C.

### Fermentation and Purification of Papaya HPL_CP-N_

HPL_CP-N_ was expressed in a 3-l BioFlo Fermenter 115 (Eppendorf, Germany) for 24 h at 25 °C with 1.5 l auto-inductive ZYM5052 broth containing 50 µg/ml kanamycin and 2.5 mM δ-aminolevulinic acid. Cultivation was inoculated with 2% (v/v) bacteria from an overnight culture. The stirrer was set to 400–800 rpm, and air was injected with 2.25 l/m (1.5 vvm) with minimum dissolved oxygen (DO) level of 30%. Cell harvesting was done as described in the “Analysis of 12-Oxododeceneoic Acid Formation” section, and the cell pellet was dissolved in binding buffer (50 mM potassium phosphate buffer pH 6 with 1 M NaCl, 40 mM imidazole, and 0.2% Triton X-100). The soluble fraction was prepared by cell disruption and centrifugation as described in the “Analysis of 12-Oxododeceneoic Acid Formation” section, and the His6-tagged HPL_CP-N_ was purified with nickel affinity chromatography using a HisTrap™ HP 5 ml column (Cytiva, USA). The soluble fraction was loaded onto the column, and then non-specific bound proteins were removed by washing with 50 mM potassium phosphate buffer pH 6 with 1 M NaCl containing 0.1% Triton X-100 and 40–100 mM imidazole. Finally, HPL_CP-N_ was eluted with 500 mM imidazole and stored at 4 °C until further use.

HPL_CP-N_ purification was monitored by SDS-PAGE (sodium dodecyl sulfate polyacrylamide gel electrophoresis) with Coomassie Brilliant Blue R250 staining, and the HPL_CP-N_ band was verified by Western blotting. Proteins were transferred onto a PVDF (polyvinylidene difluoride) membrane and incubated with the monoclonal AP-conjugated Anti-His (C-term) antibody (Invitrogen, Thermo Fisher Scientific, USA, AB_2556555) in a 1:2000 dilution. Histidine-tagged proteins were visualized with alkaline phosphatase (New England Biolabs GmbH, Germany).

### Preparation of Fatty Acid Hydroperoxides

Linoleic acid was prepared from safflower oil by alkaline hydrolysis and enrichment by urea crystallization as described before [11]. Linoleic acid was then diluted in 2.5 l of cold 50 mM sodium borate buffer (pH 9.5) to a final concentration of 1 mM. The peroxidation reaction was started by addition of 15 mg LOX-1 and performed for 1 h under stirring and a constant flow of 400 ml/min pure oxygen at 4 °C. The solution was acidified to pH 3.5 with HCl before adding an equivalent volume of ethyl acetate. The organic phase was separated and washed with water before evaporating residual solvent under vacuum. Final HPODE content and regioisomeric ratio were determined photometrically at 234 nm and by HPLC analysis as described before [11].

### Characterization of Papaya HPL_CP-N_

Enzymatic activity was determined photometrically with a UV-3100PC spectrophotometer from VWR (Germany). If not noted otherwise, 10 µl HPL_CP-N_ in an appropriate dilution was mixed with 990 µl 50 mM potassium phosphate buffer pH 6 with 1 M NaCl and 40 µM 13S-HPODE. The decrease of absorption was measured for 300 s at 22 °C at 234 nm, correlating to the decline of the conjugated double bond system of 13S-HPODE. The activity was measured in units. One unit is defined by the amount of enzyme that catalyzes 1 µmol substrate per minute. Volumetric activity was calculated using an extinction coefficient of 23,000 M^−1^·cm^−1^. Specific activity was calculated after determination of protein concentration according to the method of Bradford with Coomassie Brilliant Blue G250 staining against a bovine serum albumin calibration curve. All measurements were performed in triplicate, and the average value and the standard deviation were calculated with Microsoft Excel.

For determination of the pH profile of purified HPL_CP-N_, pH values from 6 to 9 were tested. The kinetic parameters K_m_ and v_max_ of HPL_CP-N_ were analyzed with substrate concentrations ranging from 5 to 100 µM 13S-HPODE and 13S-HPOTE. The volumetric activity was measured in triplicate with Microsoft Excel, and the kinetic parameters with standard errors were calculated through nonlinear regression with the program GraphPad Prism 6.05.

The molecular weight of purified HPL_CP-N_ was determined with size exclusion chromatography using a Superdex™ 200 Increase 10/300 column (Cytiva, USA). The column was equilibrated with 50 mM potassium phosphate buffer pH 7 with 0.5 M NaCl pH 7 and 0.1% Triton X-100. The column was calibrated with the gel filtration markers kit ranging from 29,000 to 700,000 Da (Sigma-Aldrich, USA), and the distribution coefficient K_AV_ was calculated as

$${K}_{AV}= \frac{{V}_{e}-{V}_{0}}{{V}_{c}-{V}_{0}}$$

where *V*_e_ is elution volume, *V*_0_ void volume, and *V*_c_ volume of the column.

HPL_CP-N_ was loaded onto the column, and the molecular weight was calculated from the calibration curve of K_AV_ versus the logarithm of the protein molecular weight (*y* =  − 0.3582x + 1.04). The oligomerization state of HPL_CP-N_ was analyzed by comparing the calculated mass to the predicted monomer mass.

### Synthesis of 12-Oxododecenoic with HPL_CP-N_

Reaction mixtures of 500 µl were prepared with 5 U HPL_CP-N_ and 1 mM 13S-HPODE in 50 mM potassium phosphate buffer pH 6 with 1 M NaCl and 0.2% Triton X-100. Reaction mixtures were incubated for different time intervals up to 120 min. Reactions were terminated by adding 500 µl of 4 mg/ml sodium borohydride in 20 mM NaOH directly into the reaction vessels. Addition of alkaline sodium borohydride catalyzes the hydrogenation of 13S-HPODE, 12-oxododecenoic acid, and hexanal to the corresponding hydroxides. After 1 h of borohydride hydrogenation, the reaction mixtures were acidified to pH 2 with HCl and extracted with methyl tert-butyl ether (MTBE). All reactions were performed in triplicate. The solvent extracts were used for further GC analysis.

### One-Pot Reactions with LOX-HPL and Lipase-LOX-HPL

One-pot reactions with LOX-1 from *G. max* and purified HPL_CP-N_ were done with varying concentrations of linoleic acid dissolved in 50 mM potassium phosphate buffer pH 7.5 containing 0.5 M NaCl and 0.05% Triton X-100. 40 Units of LOX-1 were added to the linoleic acid containing buffer to a final volume of 400 µl to start the hydroperoxidation reaction. Four hundred microliters of buffer containing purified HPL_CP-N_ (20 U/ml) was either added simultaneously or after a pre-incubation of LOX-1 and then reacted for additional 15 min in the presence of both enzymes (consecutive reaction mode). All reactions were done at 22 °C in open cups with vortexing in intervals. The reactions were terminated by addition of an equal volume of alkaline sodium borohydride, and products were extracted as described in the previous section.

One-pot reactions with lipase from *P. fluorescens*, LOX-1 from *G. max,* and purified HPL_CP-N_ were done essentially as described above for the two-enzyme system with simultaneous and consecutive enzyme addition. Safflower oil corresponding to an initial concentration of 2 mM linoleic acid equivalent in a volume of 300 µl was dissolved in buffer and hydrolyzed with lipase (17.6 U/ml). Three hundred microliters of LOX-1 (100 U/ml) was added over 3 h in 12 portions of 25 µl, and after 3 h of reaction, 300 µl of HPL_CP-N_ (20 U/ml) was added. Upon addition of LOX and HPL, the reaction mixture was diluted to a final linoleic acid equivalent concentration of 0.67 mM in a volume of 900 µl. The reactions were terminated by addition of an equal volume of alkaline sodium borohydride, and products were extracted as described in the “Synthesis of 12-Oxododecenoic with HPL_CP-N_” section.

### Product Analysis by GC–MS and Quantification by GC-FID

Samples in MTBE were silylated with 20% (v/v) BSTFA-TMCS (99:1) for 1 h at 80 °C. A GC–MS-QP2020 from Shimadzu (Japan) equipped with an ERAcc-5MS column from Isera GmbH (Germany) (length: 15 m, film thickness: 0.1 µm, inner diameter 0.32 mm) was used. Product identification was performed by comparing to the reference substances 13S-HPODE, 12-oxo-9(Z)-dodecenoic acid, 12-oxo-10(E)-dodecenoic acid, hexanal, and hexanol. Samples of 1 µl were injected with a split ratio of 10, and helium was used as carrier gas. A temperature gradient starting from 40 °C was applied: 40 to 200 °C within 15 °C min^−1^, 200 to 280 °C within 5 °C min^−1^, and hold at 280 °C for 2 min. Mass spectra were obtained by electron ionization (EI), and spectra were recorded in the range of 40–500 m/z.

Product quantification was done with a GC-2100 equipped with flame ionization detector (FID) (Shimadzu, Japan) using a MTX-Biodiesel TG column (length: 14 m, film thickness: 0.16 µm, inner diameter: 0.53 mm) from Restek GmbH (Germany) and helium as carrier gas. Samples of 1 µl were injected with a split ratio of 10, and a temperature gradient starting from 40 °C was used: 40 to 175 °C with 12 °C min^−1^, 175 to 210 °C with 5 °C min^−1^, 210 to 330 °C with 25 °C min^−1^, and hold at 330 °C for 2 min. For product quantification, calibration curves were generated with the hydrogenated and silylated reference substances linoleic acid, 13S-HPODE, 12-hydroxydodecanoic acid, and hexanal.

## Results and Discussion

### Cloning of Papaya HPL and Optimization of Expression

For the development of a one-pot enzyme cascade with lipase, LOX, and HPL, high amounts of enzymes are needed. While lipases and lipoxygenases can be obtained easily in large quantities, HPLs are not commercially available due to their low stability and poor solubility in aqueous solutions. Purification of HPLs from plant materials is complicated and cost-intensive. Therefore, cloning and expression of HPL in microbial hosts are a suitable method for HPL synthesis [14, 37].

**Fig. 2:**
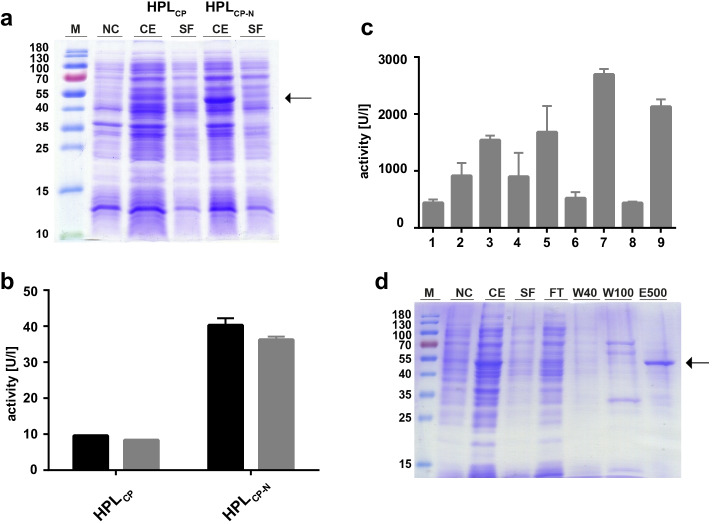
Comparison of HPL_CP_ with HPL_CP-N_ and optimization of cultivation media. **(a)** SDS-PAGE of the crude extract (CE) and soluble fraction (SF) of HPL_CP_ and HPL_CP-N_ with (M) marker protein ladder (PageRuler™ Prestained protein ladder, Thermo Fisher Scientific, USA) and (NC) negative control with empty pET-28a( +) vector. **(b)** Enzyme activity of HPL_CP_ and HPL_CP-N_ per liter cultivation medium with black bar: crude extract and gray bar: soluble fraction. **(c)** Comparison of cultivation media with 1, LB; 2, TB; 3, TB + δ-aminolevulinic acid (ALA); 4, TB + ammonium ferric citrate (Fe); 5, TB + ALA + Fe; 6, ZYM5052; 7, ZYM5052 + ALA; 8, ZYM5052 + Fe; 9, ZYM5052 + ALA + Fe. **(d)** Purification of HPL_CP-N_ by SDS-PAGE with M, marker protein ladder; NC, negative control with empty pET-28a( +) vector; CE, crude extract; SF, soluble fraction; FT, flow-through; W40, washing fraction with 40 mM imidazole; W100, washing fraction with 100 mM imidazole; and E500, HPL_CP-N_ elution fraction with 500 mM imidazole

On basis of the known sequence of industrially used guava HPL [23] (accession number AAK15070.1), we identified a related protein sequence from *C. papaya* (HPL_CP_) by BLAST analysis. The putative HPL (accession number XP_021890218.1, Fig. S1) has an identity of 66.88% compared to the guava sequence. A phylogenetic tree was drawn with known HPLs from the CYP74B and CYP74C subfamily using ClustalX and NJplot (Fig. S3). Based on the phylogenetic relations, HPL_CP_ can be assigned to the CYP74B subfamily that comprises 13 specific HPLs. The respective gene was synthesized by BioCat GmbH (Germany) and cloned into the pET-28a( +) expression vector (Fig. S1). The enzyme was expressed in *E. coli* BL21(DE3) in LB medium. After harvest, the cell pellet was dissolved in 50 mM Tris buffer pH 7 with 0.05 M NaCl and 0.2% Triton X-100 and disrupted through sonication. Only a slight protein band was visible on SDS-PAGE (Fig. 2a), and activity of the full-length protein was low with 10 units per liter cultivation medium (Fig. 2b). Therefore, we tried to enhance activity of HPL_CP_ by N-terminal truncation. The non-conserved N-terminal sequence of HPL_CP_ was identified in a multiple sequence alignment with several known HPL sequences with ClustalW (Fig. S2) and removed through PCR-based subcloning (Fig. S1). After expression of HPL_CP-N_ in *E. coli* BL21(DE3), a fourfold increase of activity was obtained in comparison to the full-length enzyme (Fig. 2b). A protein band was visible on SDS-PAGE at 53 kDa in the crude extract (CE) indicating an improved expression of the truncated enzyme (Fig. 2a). However, no protein band was visible in the soluble fraction (SF). Thus, it seems that most of the HPL protein was not present in a solubilized form. Therefore, the solubilization buffer was optimized starting from the initially used 50 mM Tris buffer pH 7 with 0.05 M NaCl (set to 100% relative activity). The effects of different buffer components, pH values, salts, and detergents on the final enzyme activity in the CE and SF were analyzed by one factor at a time variation (OFAT, Fig. S4a–d). Highest activity in the CE was obtained at a pH of 6 (Fig. S4a), in the presence of potassium phosphate as buffering substance (Fig. S4b) and by addition of 1 M NaCl (Fig. S4c). In all experiments, the activity of the SE was significantly lower than that of the CE. An increase in activity of the SE was obtained by adding the detergent Triton X-100 (Fig. S4d). Combination of the best conditions (50 mM potassium phosphate buffer pH 6 with 1 M NaCl and 0.2% Triton X-100) increased the activity in the CE and SF more than eight-fold in comparison to the initial 50 mM Tris buffer pH 7 with 50 mM NaCl (Fig. S4e). Next, different cultivation media (Table S1) were analyzed for higher HPL_CP-N_ expression. A significant increase in HPL expression was obtained by using the auto-inductive ZYM5052 medium with δ-aminolevulinic acid (Fig. 2c).

Summarizing, His6-tagged papaya HPL was successfully cloned into pET-28a( +) expression vector and expressed in *E. coli* BL21(DE3) as full-length and N-terminally truncated form. Though comparatively high HPL activity was reported for papaya fruit extracts [23], the enzyme has not been expressed recombinantly before. The expression level of full-length HPL_CP_ was low on SDS-PAGE, while it increased in the expression of the truncated HPL_CP-N_. The reason for this in unclear, and we suggest that the deletion of the hydrophobic N-terminus must play a role due to the fact that the expression increased upon truncation. The activity of papaya HPL in the soluble fraction could be increased from initial 10 U/l with the full-length enzyme to 2700 U/l in shake flask cultures with the truncated HPL under optimized expression and solubilization conditions. As described for alfalfa or guava HPL [25, 31], solubility of full-length HPL proteins seems to be a common problem resulting in low or no HPL activity. For papaya full-length HPL, we could only measure low activity as well, and since no corresponding protein band was visible on SDS-PAGE, it can be concluded that HPL expression of the full-length HPL_CP_ was rather low. Expression of the truncated HPL resulted in higher activity and a visible protein band on SDS-PAGE. Therefore, we suggest that the hydrophobic N-terminus negatively influences the expression level of HPL_CP_. Addition of δ-aminolevulinic acid as heme precursor leads to an increase of soluble HPL expression in accordance to results obtained for beet, bell pepper, and olive HPL [38–40]. Additionally, buffer optimization had a significant effect on increasing the activity of HPL_CP-N_ in the crude extract (CE) as well as in the soluble fraction (SF). A major factor was the addition of detergent Triton X-100, which solubilized HPL_CP-N_ and increased activity in the SF. In accordance to our results, solubility and activity increase in the presence of detergents was reported for barley and barrel medic HPLs [41, 42]. Although a more than 100-fold activity increase was obtained by optimization of cultivation and solubilization buffer, Bradford staining did not exhibit a HPL_CP-N_ protein band in the SF (Fig. 2d). Thus, further optimization of expression and solubilization may improve volumetric HPL activity.

### Purification and Biochemical Characterization of HPL_CP-N_

HPL_CP-N_ was expressed under optimized conditions in 1.5 l scale to obtain sufficient amount of enzyme for downstream processing and characterization. In a typical process more than 7000 units, HPL_CP-N_ was obtained after cell harvest, cell disruption, and solubilization in buffer. After centrifugation, a specific activity of 1.27 U/mg was obtained in the soluble fraction (Table 2). Purification of the His6-tagged HPL_CP-N_ was done with nickel affinity chromatography, and the purification process was analyzed by SDS-PAGE (Fig. 2d). A protein band was detected after affinity chromatography indicating significant HPL enrichment. Western blot was performed with an Anti-His antibody, confirming the identity of the His-tagged HPL_CP-N_ protein (Fig. S5). The specific activity of HPL_CP-N_ was increased more than 15-fold in the eluate fraction of the affinity chromatography (Table 2).

**Table 2:** Fermentation and purification of HPL_CP-N_. BL21(DE3)/ pET**-**28a::His*hpl*_CP-N_ was cultivated in a 3 l bioreactor with a volume of 1.5 l ZYM5052 + δ-aminolevulinic acid, and cells were harvested after 24 h of cultivation

	**Total activity [U]**	**Volume [ml]**	**Volumetric activity [U/ml]**	**Protein concentration [mg/ml]**	**Specific activity [U/mg]**
Crude extract	7174	600	11.96	10.41	1.15
Soluble fraction	5722	600	9.54	7.53	1.27
Eluate	2447	120	20.39	1.12	18.21

According to gel filtration analysis, HPL_CP-N_ has a calculated molecular weight of 225.9 kDa (Fig. S6). Based on the monomer molecular weight of 53 kDa from the truncated and His-tagged sequence, it can be presumed that HPL_CP-N_ appears as a tetramer, which correlates with previous analyses of guava and sunflower HPL [23, 43]. A pH analysis in the range of 6–9 revealed that the enzyme shows highest activity under slightly acidic conditions but retains approximately 40% of its maximum activity at pH 9 (Fig. S7). The kinetic parameters of the affinity chromatography enriched HPL_CP-N_ were measured at pH 6 and calculated with GraphPad Prism 6.05 (Table 3, Fig. S8). HPL_CP-N_ has a K_m_ value of 140 µM for linoleic acid hydroperoxide (13S-HPODE) and 150 µM for linolenic acid hydroperoxide (13S-HPOTE) suggesting a relative similar substrate affinity. However, HPL_CP-N_ exhibits a 1.55 fold higher catalytic efficiency (k_cat_/K_m_) with 13S-HPOTE as substrate in comparison to 13S-HPODE, which indicates a weak preference of HPL_CP-N_ towards 13S-HPOTE. In contrast, the truncated form of olive HPL showed a 22.5 fold increase of turnover with 13S-HPOTE compared to 13S-HPODE and a 5.5 fold higher catalytic efficiency (2.36 × 10^6^ versus 0.43 × 10^6^ s^−1^·M^−1^) [40]. Similarly, HPLs from *Medicago truncatula* and *Solanum tuberosum* showed a clear preference for 13S-HPOTE [42, 44]. The comparably high catalytic activity of HPL_CP-N_ with the substrate 13S-HPODE is beneficial for the cascade with lipase and LOX starting from safflower oil rich in linoleic acid.

**Table 3:** Kinetic parameters of HPL_CP-N_ calculated with GraphPad Prism 6.05 with data from Fig. S8

Substrates	K_m_ [µM]	V_max_ [µM·s^−1^]	k_cat_ [s^−1^]	k_cat_/K_m_ [s^−1^·M^−1^]
13S-HPODE	140 ± 30	1452 ± 224	382	2.73 × 10^6^
13S-HPOTE	150 ± 40	2408 ± 487	634	4.23 × 10^6^

### Analysis of 12-Oxododeceneoic Acid Formation

**Fig. 3:**
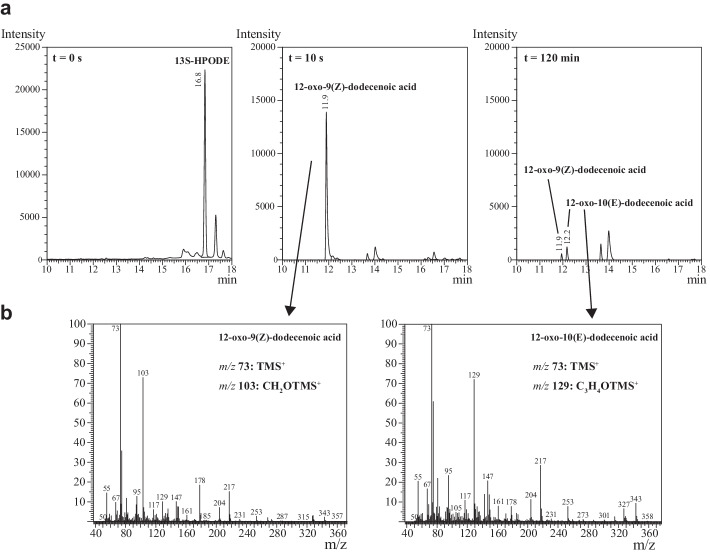
**(a)** GC-FID chromatograms of substrate 13S-HPODE and after transformation with HPL_CP-N_ (soluble fraction, 22 °C) for 10 s and 120 min including **(b)** GC–MS fragmentation pattern of peaks at 11.9 min and 12.2 min retention time. Full GC-FID chromatograms including hexanal peaks and reference GC–MS spectra of 12-oxo-9(Z)-dodecenoic acid, 12-oxo-10(E)-dodecenoic acid, and hexanal are shown in Figures S9 and S10

HPL-catalyzed synthesis of green note aldehydes often neglected the by-product 12-oxo-9(Z)-dodecenoic acid, though the compound may serve as polymer precursor. Reaction mixtures containing 1 mM 13S-HPODE were incubated up to 120 min with the soluble fraction of *E. coli* expressing HPL_CP-N_. Product formation was analyzed after sodium borohydride reduction and extraction. GC–MS peak assignment and GC quantification revealed rapid disappearance of 13S-HPODE and formation of a new peak at 11.9 min (Fig. 3). After 120 min, the peak at 11.9 min decreased significantly, and a new peak appeared at 12.2 min. The major signals were *m/z* 73 and 103 for the peak at 11.9 min and *m/z* 73 and 129 for the peak at 12.2 min (Fig. 3). This characteristic pattern of the silylated compounds points to the release of 12-oxo-9(Z)-dodecenoic acid and the consecutive isomerization to 12-oxo-10(E)-dodecenoic acid (traumatin). Comparative analysis with reference substances verified the fragmentation patterns (Fig. S10), which were also found by Noordermeer et al. [45].

**Fig. 4:**
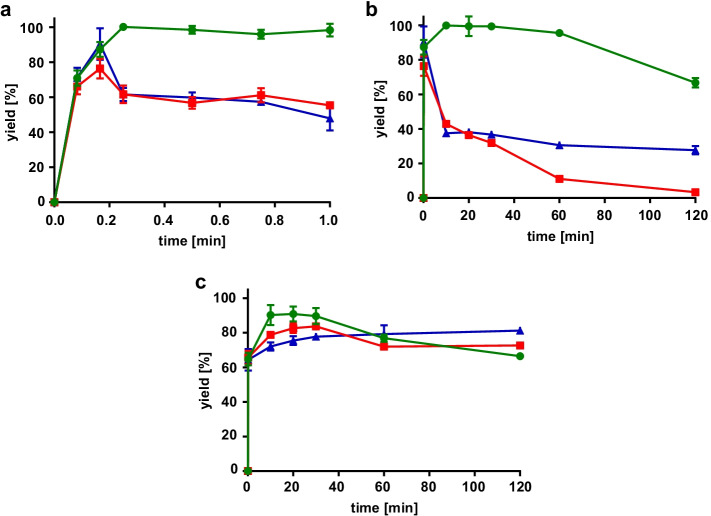
Monitoring of 12-oxo-9(Z)-dodecenoic acid **(a + b)** and hexanal **(c)** from HPL_CP-N_ catalysis with 1 mM 13S-HPODE substrate at pH 6. Green circle = 10 U/ml purified HPL_CP-N_ at 22 °C; red box = 10 U/ml soluble fraction of HPL_CP-N_ at 22 °C; blue triangle = 10 U/ml soluble fraction of HPL_CP-N_ on ice; unfilled red box + dotted red line = 12-oxo-10(E)-dodecenoic acid formation with 10 U/ml soluble fraction of HPL_CP-N_

In time-course experiments, it became apparent that the concentration of 12-oxo-9(Z)-dodecenoic acid reached its maximum after 10 s and started to decrease rapidly (Fig. 4a and b). Up to 0.1 mM of the isomerization product 12-oxo-10(E)-dodecenoic acid (traumatin) was detected after 120 min (Fig. 4b). Incubation on ice slowed down the decrease of 12-oxo-9(Z)-dodecenoic acid, but could not prevent consecutive reactions. The second reaction product hexanal proved to be significantly more stable, though a slight concentration decrease was monitored (Fig. 4c).

**Fig. 5:**
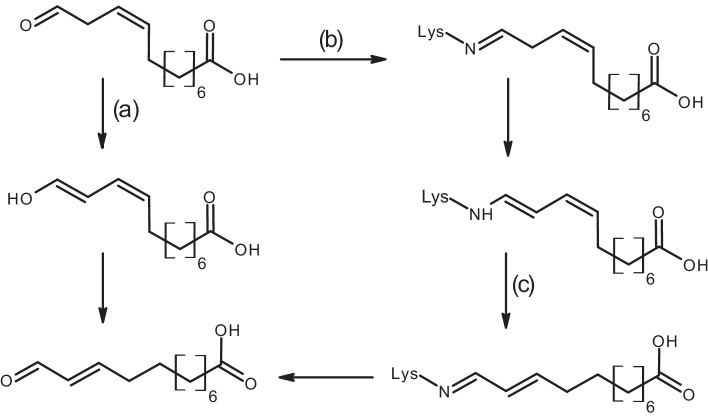
Possible routes explaining 12-oxo-9(Z)-dodecenoic acid disappearance and 12-oxo-10(E)-dodecenoic acid (traumatin) formation with **(a)** keto-enol tautomerism, **(b)** Schiff’s base formation, and **(c)** imine-enamine tautomerism

The isomerization product traumatin was described before for guava HPL [18]. Grechkin and Hamberg proposed the enol to be formed upon cleavage of the hemiacetal intermediate leading to either 9(Z)- or 10(E)-oxododecenoic acid tautomers [18]. Surprisingly, formation of traumatin was only detected in traces with purified HPL_CP-N_, and stability of 12-oxo-9(Z)-dodecenoic acid was significantly higher (Fig. 4b). In our opinion, the differences in traumatin formation indicate secondary isomerization processes not specifically related to HPL (Fig. 5). Keto-enol tautomerism followed by double-bond shifting leads to traumatin formation. The formation of Schiff bases and isomerization of the double bond system via an imine-enamine tautomerism may be responsible for additional release of traumatin. In the presence of protein-rich crude extracts, an overall loss of reaction products was monitored suggesting Schiff base formation with, e.g., proteinogenic lysine residues. Thus, utilization of purified enzyme and rapid extraction seem to be necessary to quantitatively isolate 12-oxo-9(Z)-dodecenoic acid. Similarly in alfalfa HPL preparations, purified from its seeds, 12-oxo-10(E)-dodecenoic acid was found in the crude fraction, while 12-oxo-9(Z)-dodecenoic acid was the main product upon HPL purification [45]. Noordermeer et al. proposed a 3Z:2E-enal isomerase as isomerization factor, whereas our experiments with recombinant HPL strongly point to non-enzymatic isomerization. Nevertheless, a 3Z:2E-enal isomerase acting on hexenal was recently found in plants and cloned from cucumber [46, 47].

### One-Pot Enzyme Cascade of HPL_CP-N_ Coupled with LOX-1 and Lipase

The development of enzyme cascades possesses significant advantages over successive reactions including a shift of equilibria without the need for isolation of intermediates [48, 49]. In our previous work, we developed a cascade reaction for 13S-HPODE synthesis from safflower oil utilizing lipase from *P. fluorescens*, LOX-1 from soybeans, and catalase for in situ oxygen generation [11]. Now we wanted to couple lipase and LOX-1 reaction with HPL_CP-N_ to prove the concept of a one-pot synthesis of 12-oxo-9(Z)-dodecenoic acid and hexanal from safflower oil. In contrast, the industrial process for volatile aldehyde production uses plant extracts in separate reactors for each reaction step [13].

**Fig. 6:**
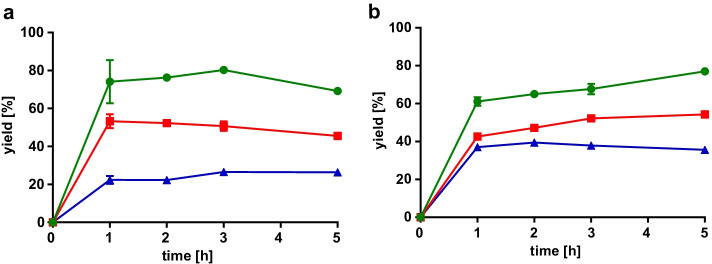
Time-dependent one-pot enzymatic reaction with LOX-1 and HPL_CP-N_. **(a)** LOX-1 reactions were conducted from 1–5 h with 1 (green circle), 2.5 (red box), and 5 (blue triangle) mM linoleic acid, and yield of 13S-HPODE was determined after extraction of the reaction mixtures. **(b)** LOX-1 reactions were carried out as in (a), and then an equal volume of HPL_CP-N_ was added for another 15 min, and 12-oxo-9(Z)-dodecenoic acid was monitored after extraction of reaction mixtures. Yield is given as percentage [%] of 12-oxodoecenoic acid based on the 13S-HPODE contents from (a)

**Fig. 7:**
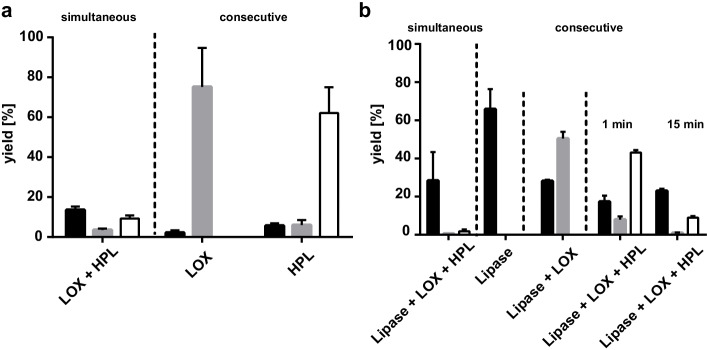
Comparison of simultaneous or consecutive enzyme addition with **(a)** one-pot reaction with LOX-1 and HPL_CP-N_ and **(b)** one-pot reaction with Amano lipase from *P.* *fluorescens*, LOX-1, and HPL_CP-N_. Black bars, linoleic acid; gray bars, 13S-HPODE; and white bars, 12-oxo-9(Z)-dodecenoic acid as yield based on the amount of starting material linoleic acid to a final concentration of 0.5 mM (a) or safflower oil with linoleic acid equivalent to a final concentration of 0.67 mM (b)

First, LOX-1 reaction was combined with the HPL_CP-N_ reaction in a one-pot experimental set-up. The pH optimum of LOX-1 is pH 9, whereas HPL_CP-N_ is most active at pH 6, yet both enzymes exhibited sufficient activity at pH 7.5 (Fig. S7), and 13-regioselectivity of LOX-1 is still around 90% at pH 7.5 [11]. Linoleic acid with starting concentrations between 1 and 5 mM was pre-incubated with LOX-1 for 1 to 5 h, and then HPL_CP-N_ was added for 15 min. Reaction mixtures were extracted and analyzed on GC. Small-scale reactions were conducted without active oxygen supply, which caused O_2_ depletion at higher linoleic acid concentrations leading to a decrease of 13S-HPODE yield (Fig. 6a). Transformation of the 13S-HPODE to 12-oxo-9(Z)-dodecenoic acid with HPL_CP-N_ yielded up to 80% transformation for the lowest linoleic acid concentration applied (Fig. 6b). At higher initial linoleic acid concentrations, HPL_CP-N_ transformation was lower pointing to an incipient substrate inhibition, which was also described for other HPLs [14]. The comparison of simultaneous LOX-1 and HPL_CP-N_ reaction for 3 h with consecutive addition of HPL_CP-N_ after 3 h revealed that product recovery rates were low in simultaneous reaction mode (Fig. 7a). This observation was expected from our product incubation studies described in the previous section. In contrast, a good overall yield of 62% 12-oxo-9(Z)-dodecenoic acid was achieved by consecutive enzyme addition starting from 1 mM linoleic acid and HPL_CP-N_ for 15 min.

Additionally, the combination of *P. fluorescens* lipase with LOX-1 and HPL_CP-N_ was tested with simultaneous and consecutive addition of the enzymes (Fig. 7b). As control reactions, the transformation of safflower oil with lipase alone and with lipase and LOX was analyzed to monitor oil hydrolysis and hydroperoxidation. Again, simultaneous addition of all enzymes resulted in extremely low product recovery of less than 5%. Lipase-catalyzed hydrolysis of safflower oil equivalent to 2 mM linoleic acid yielded 66% hydrolysis, and around 2/3 of the liberated fatty acids were peroxidized by LOX-1. In contrast to the LOX-HPL two-enzyme system, significantly lower yields of 12-oxo-9(Z)-dodecenoic acid were obtained upon 15 min of HPL_CP-N_ reaction. This observation indicates a rapid product transformation caused by addition of the lipase preparation. HPL_CP-N_ reactions below 1 min proved to be sufficient for quantitative 13S-HPODE transformation (Fig. 4). Thus, the reaction of the three-enzyme system was terminated by rapid extraction 1 min after addition of HPL_CP-N_. With this methodology, a final yield of 43% 12-oxo-9(Z)-dodecenoic acid was obtained from safflower oil in the one-pot enzyme cascade (Fig. 7b).

## Conclusions

Hydroperoxide lyase from papaya was cloned, functionally expressed as N-terminally truncated enzyme, purified and characterized biochemically. HPL_CP-N_ was successfully applied for the synthesis of 12-oxo-9(Z)-dodecenoic acid from 13S-HPODE, and a one-pot enzyme cascade in combination with lipase and LOX-1 starting from safflower oil was established. Instability of the reactive product needs proper control of reaction conditions, and further research is needed to optimize process conditions for larger scale synthesis of 12-oxo-9(Z)-dodecenoic acid.

## Availability of Data

Data are available on request.

## Supplementary Information

Below is the link to the electronic supplementary material.

Supplementary file1 (PDF 841 kb)
