# Supplementary material for: Synthesis of Polymer Precursor 12-Oxododecenoic Acid Utilizing Recombinant Papaya Hydroperoxide Lyase in an Enzyme Cascade
Source: Appl Biochem Biotechnol. 2022 Jul 29;194(12):6194–212. doi: 10.1007/s12010-022-04095-0 (PMC9708767; doi:10.1007/s12010-022-04095-0)
Supplement: Supplementary file 1 — Supplementary file1 (PDF 841 kb) [file 12010_2022_4095_MOESM1_ESM.pdf]

Supplementary data:

# Synthesis of polymer precursor 12-oxododecenoic acid utilizing recombinant papaya hydroperoxide lyase in an enzyme cascade

Anna Coenen[1], Valentin Gala Marti[1], Kira Müller[1], Maria Sheremetiev[1], Lorenzo Finamore[1], Ulrich Schörken[1*]

[1] TH Köln – Campus Leverkusen, CHEMPARK, Kaiser-Wilhelm-Allee, 51368 Leverkusen, Germany
* Correspondence: ulrich.schoerken@th-koeln.de; 0049-214-32831-4610

**>HPL$_{CP}$**

MMMKLMNISPTMSSPSSPPSSSPLASNSISTPPSSALPLRTIPGSYGWPLLGPLSDRLDYFWFQG
PETFFRKRMEKNKSSVFRTNVPPSFPFFLDVNPNVIAVLDVKSFSHLFDLEIVEKKDVLVGSFV
PSTRFTGDVRVGVYLDTAEPKHSEVKNLTMELLQRGSKVWQSELLSNLDKMWDMVEATVA
EKGKATYLGPLQQCIFNFIMKALAGIDPAVSPQIANSGYIMLDRWLFLQLLPTVNIGILQPLEEI
FLHSWAYPFFLVRNDYKNLYDFIKQNGKEVLQIAETKFGLTEEETIHNLLFVIGFNAFGGFSVF
LPSLLDAISSDQTGLQDKLKKEVREHSVPGSGLDFETMSKMELVKSVVYEALRFKPPVPTQYG
RARKDFRLTSHDSVYDIKKGELLCGFQPLVMRDPEVFDEPEKFKPDRFLGEGSKLLSYLYWS
NGPQTGSPSESNKQCAAKEVVPLTACLVVAHLFLRYEKISGGSGSITALEKTKHHHHHH*

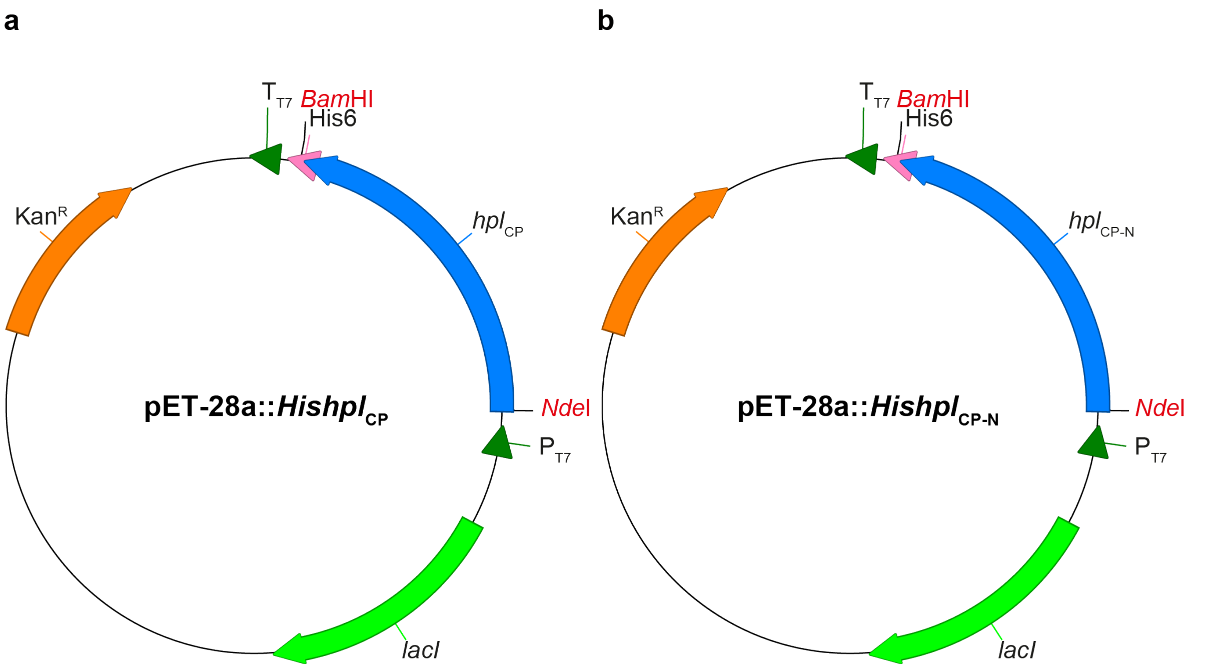


**Fig. S1** Top: Protein sequence of full length and N-terminally truncated HPL from *Carica papaya* (truncation area coded in red); bottom: Vector graphics of full-length **(a)** and truncated papaya HPL **(b)** with C-terminal hexahistidine tag

**Table S1** Composition of cultivation media

| Component | Final Concentration |
|---|---|
| **LB** | |
| Yeast extract | 5 g/l |
| Tryptone | 10 g/l |
| NaCl | 10 g/l |
| **TB** | |
| Yeast extract | 24 g/l |
| Tryptone | 20 g/l |
| Glycerol | 4 ml/l |
| Phosphate buffer | 0.017 M $KH_2PO_4$, 0.072 M $K_2HPO_4$ |
| **ZYM5052** | |
| ZY | 10 g/l Tryptone, 5 g/l Yeast extract |
| $MgSO_4$ | 2 mM |
| 1000 x Trace metal mix | 10 mM $FeCl_3$, 4 mM $CaCl_2$, 2 mM $MnCl_2$, 2 mM $ZnSO_4$, 0.4 mM $CoCl_2$, 0.4 mM $CuCl_2$, 0.4 mM $NiCl_2$, 0.4 mM $Na_2MoO_4$, 0.4 mM $Na_2SeO_3$, 0.4 mM $H_3BO_3$ |
| 5052 | 0.5 % Glycerol, 0.05 % Glucose, 0.2 % Lactose |
| Buffer | 25 mM $Na_2HPO_4$, 25 mM $KH_2PO_4$, 50 mM $NH_4Cl$ |

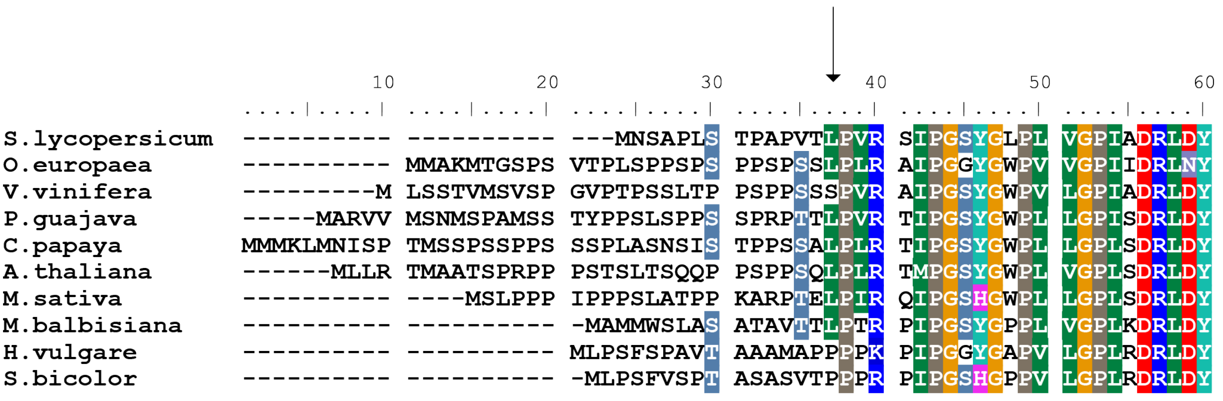


**Fig. S2** Multiple alignment of HPL sequences using ClustalW. The arrow marks the start amino acid of the truncated *Carica papaya* HPL

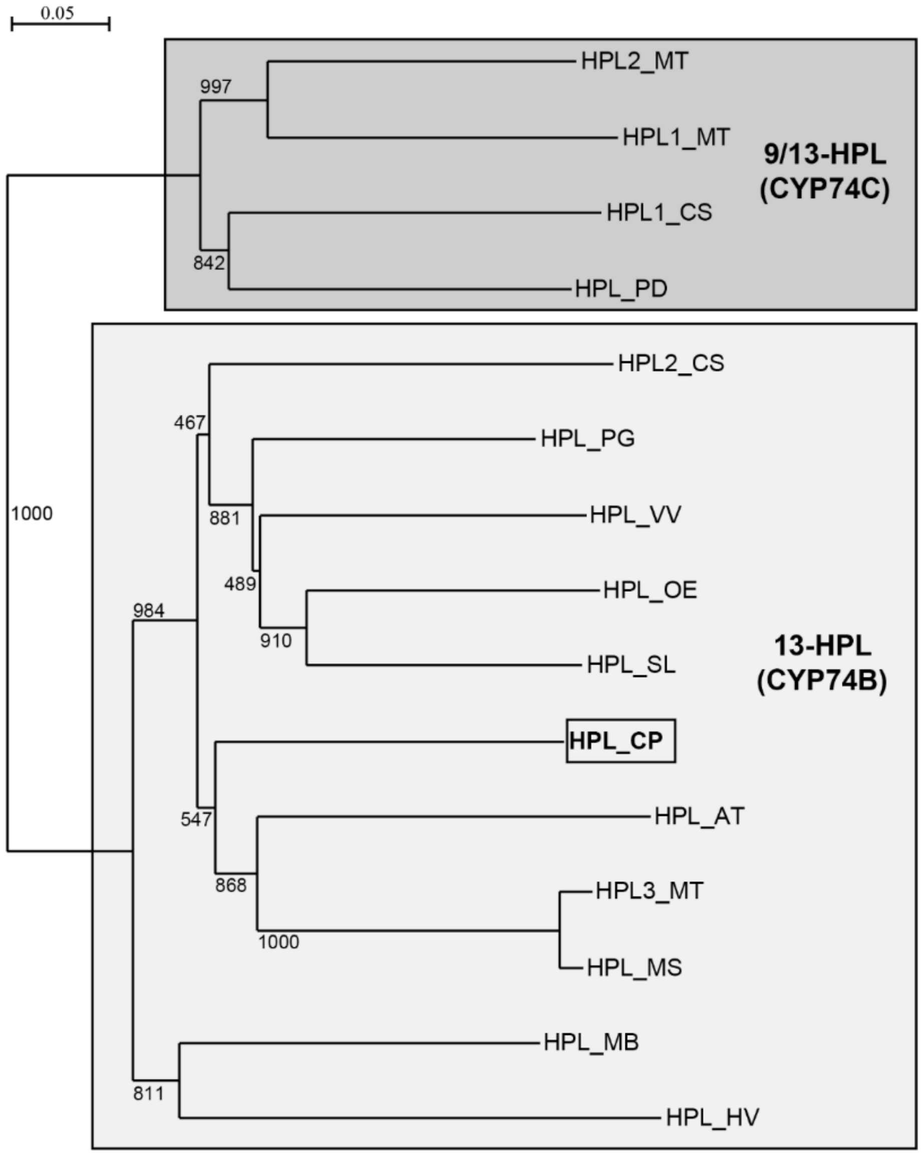


**Fig. S3** Phylogenetic tree of HPL sequences. A neighbor-joining tree was created using ClustalX and NJplot and the bootstrap value was set to 1000. The following HPLs with respective accession numbers were incorporated: MT: *Medicago truncatula* (1: CAC86898.1, 2: CAC86899.1, 3: AAY30368.1); CS: *Cucumis sativus* (1: AHC08715.1, 2: XP_004144503.1); PD: *Prunus dulcis* (CAE18065.1); PG: *Psidium guajava* (AAK15070.1); VV: *Vitis vinifera* (NP_001268011.1); OE: *Olea europaea* (ACD43482.1); SL: *Solanum lycopersicum* (NP_001234420.2); CP: *Carica papaya* (XP_021890218.1); AT: *Arabidopsis thaliana* (AAC69871.1); MS: *Medicago sativa* (CAB54847.1); MB: *Musa balbisiana* (THU49863.1) and HV: *Hordeum vulgare* (CAC82980.1).

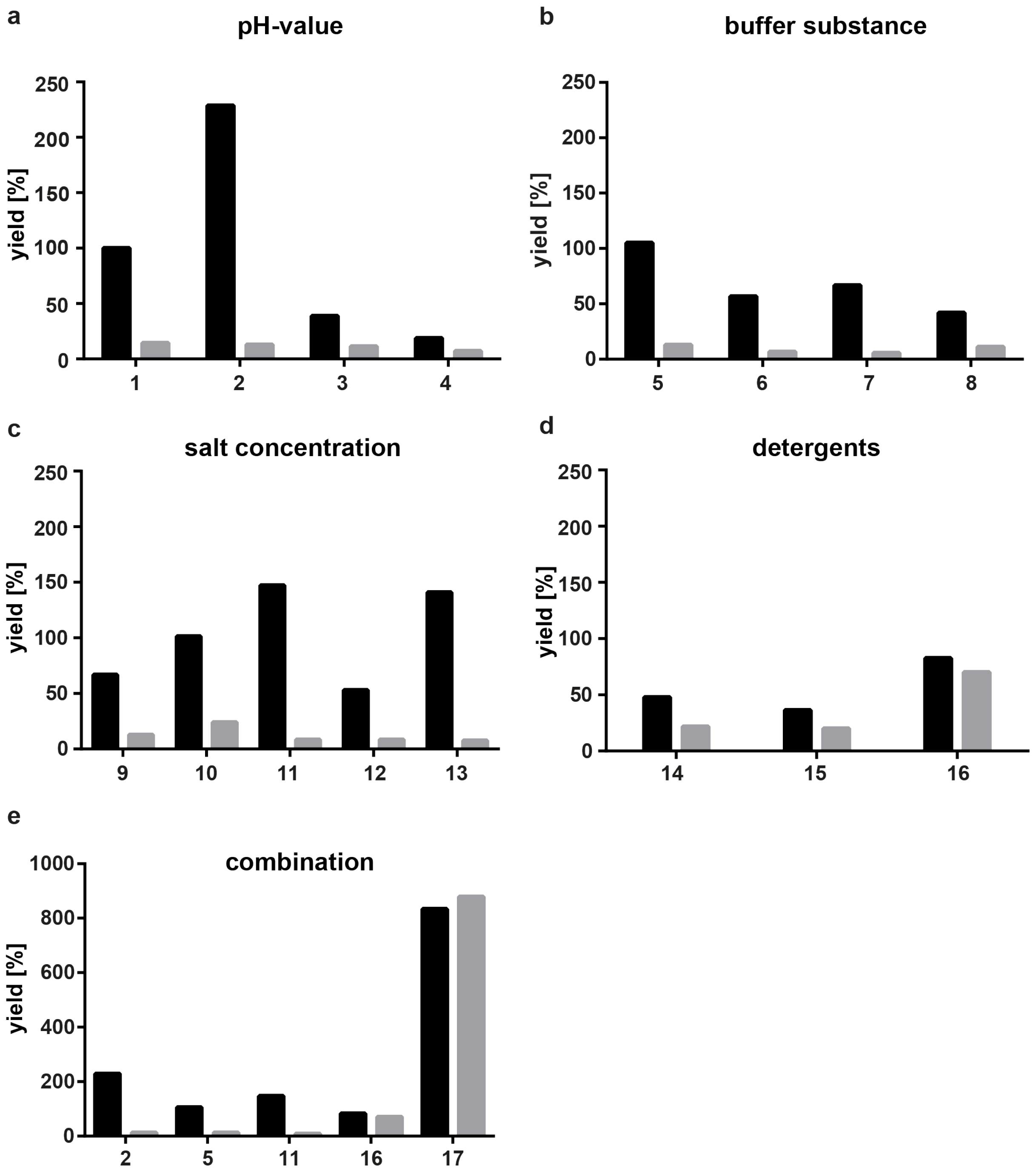


**Fig. S4** Buffer optimization for solubilization of $HPL_{CP\text{-}N}$. The HPL activity in the crude extract of the initially used 50 mM Tris buffer pH 7 with 0.05 M NaCl (buffer 1) was set to 100 %. In each step one component was changed: **(a)** pH values in the presence of 0.05 M NaCl with (1) 50 mM Tris pH 7, (2) 50 mM potassium phosphate pH 6, (3) 50 mM Tris pH 8 and (4) 50 mM Tris pH 9; **(b)** Buffer substances in the presence of 0.05 M NaCl at pH 7 with (5) 50 mM potassium phosphate, (6) 50 mM HEPES, (7) 50 mM MOPS and (8) 50 mM Bis-Tris; **(c)** Salt concentrations in the presence of 50 mM Tris with (9) no salt, (10) 0.5 M NaCl, (11) 1 M NaCl, (12) 0.1 M KCl and (13) 1 M KCl; **(d)** Detergents in the presence Tris pH 7 and 0.05 M NaCl with (14) 0.2 % Brij®, (15) 0.2 % Tween-20 and (16) 0.2 % Triton X-100; **(e)** The best conditions of (a) – (d) (buffers 2, 5, 11 and 16) were combined to (17) containing 50 mM potassium phosphate pH 6 with 1 M NaCl and 0.2 % Triton X-100;. Black bars: crude extract, grey bars: soluble fraction.

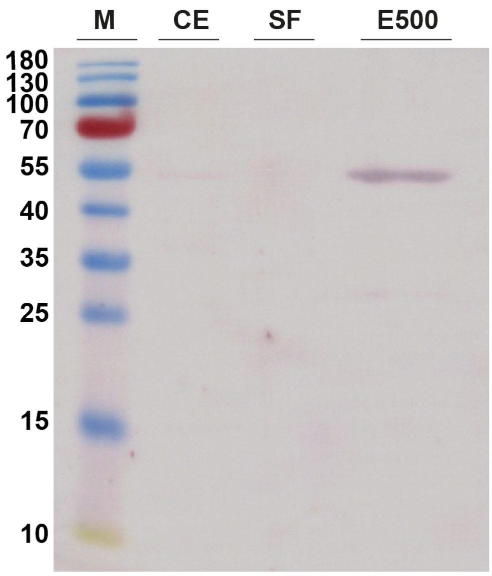


**Fig. S5** Western Blot of $HPL_{CP-N}$ purification with monoclonal AP-conjugated Anti-His (C-term) antibody; M: marker protein ladder, CE: crude extract, SF: soluble fraction and E500 purified elution fraction with 500 mM imidazole

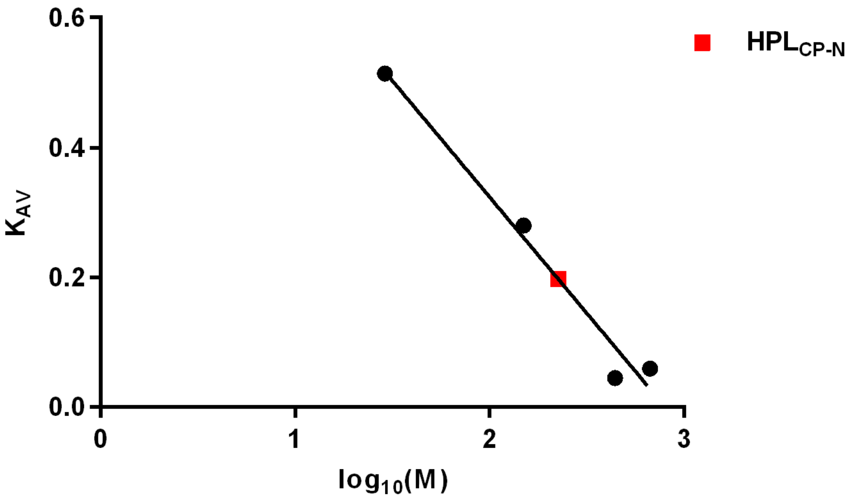


**Fig. S6** Molecular weight determination of $HPL_{CP-N}$ with gel filtration column Superdex™ 200 Increase 10/300 column (Cytiva, USA) with gel filtration markers kit for protein molecular weights 29,000-700,000 Da (Sigma-Aldrich, USA). A calibration curve was drawn with the distribution coefficient $K_{AV}$ ($K_{AV} = \frac{V_e - V_0}{V_c - V_0}$) versus the logarithm of protein molecular weight. The calculated formula is y = -0.3582x + 1.04. $V_e$ = elution volume; $V_0$ = void volume and $v_c$ = volume of the column

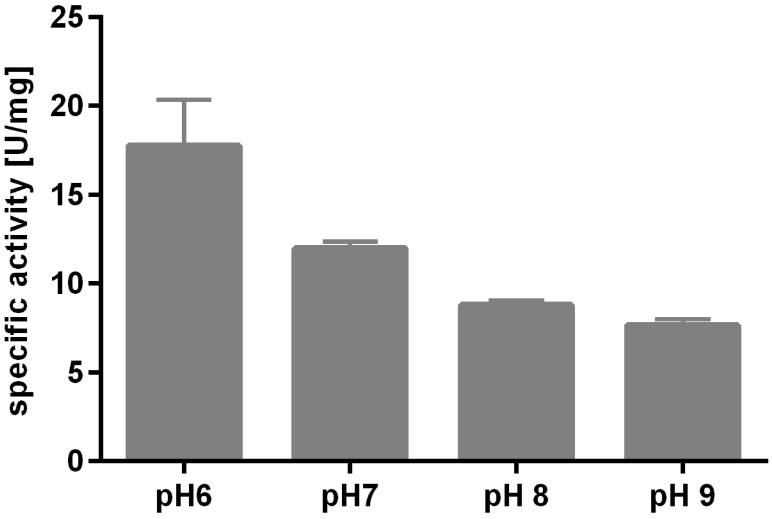


**Fig. S7** Activity profile of purified $HPL_{CP-N}$ in dependence of pH value of reaction buffer

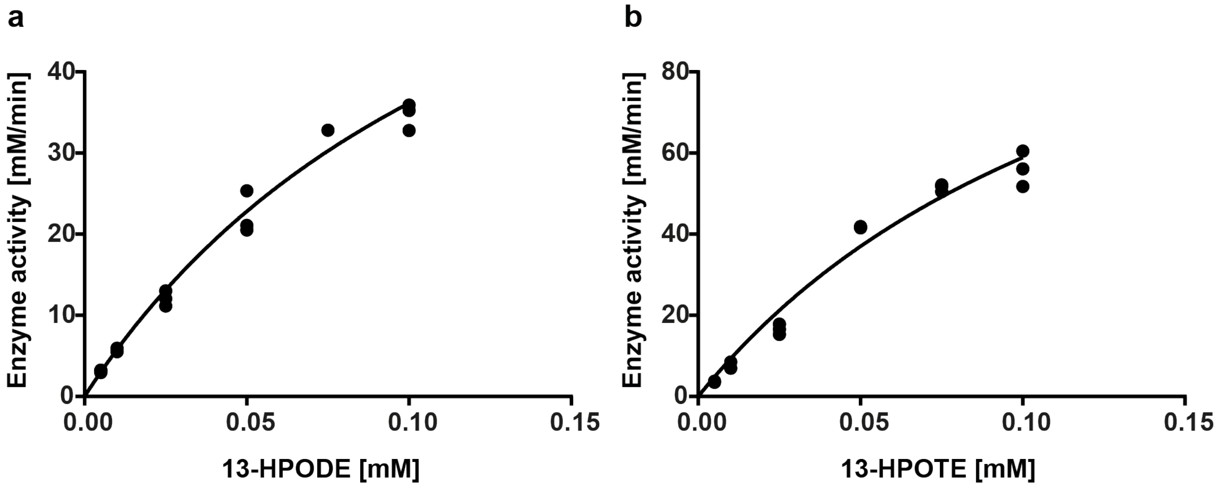


**Fig. S8** Diagrams of initial enzyme activity in relation to substrate concentration of 13S-HPODE **(a)** and 13S-HPOTE **(b)** of the enriched $HPL_{CP-N}$ after affinity chromatography. Curves were calculated with GraphPad Prism 6.05 and used for determination of kinetic constants $K_M$ and $k_{cat.}$ No correction factors to compensate for impurities of the enzyme preparation were applied.

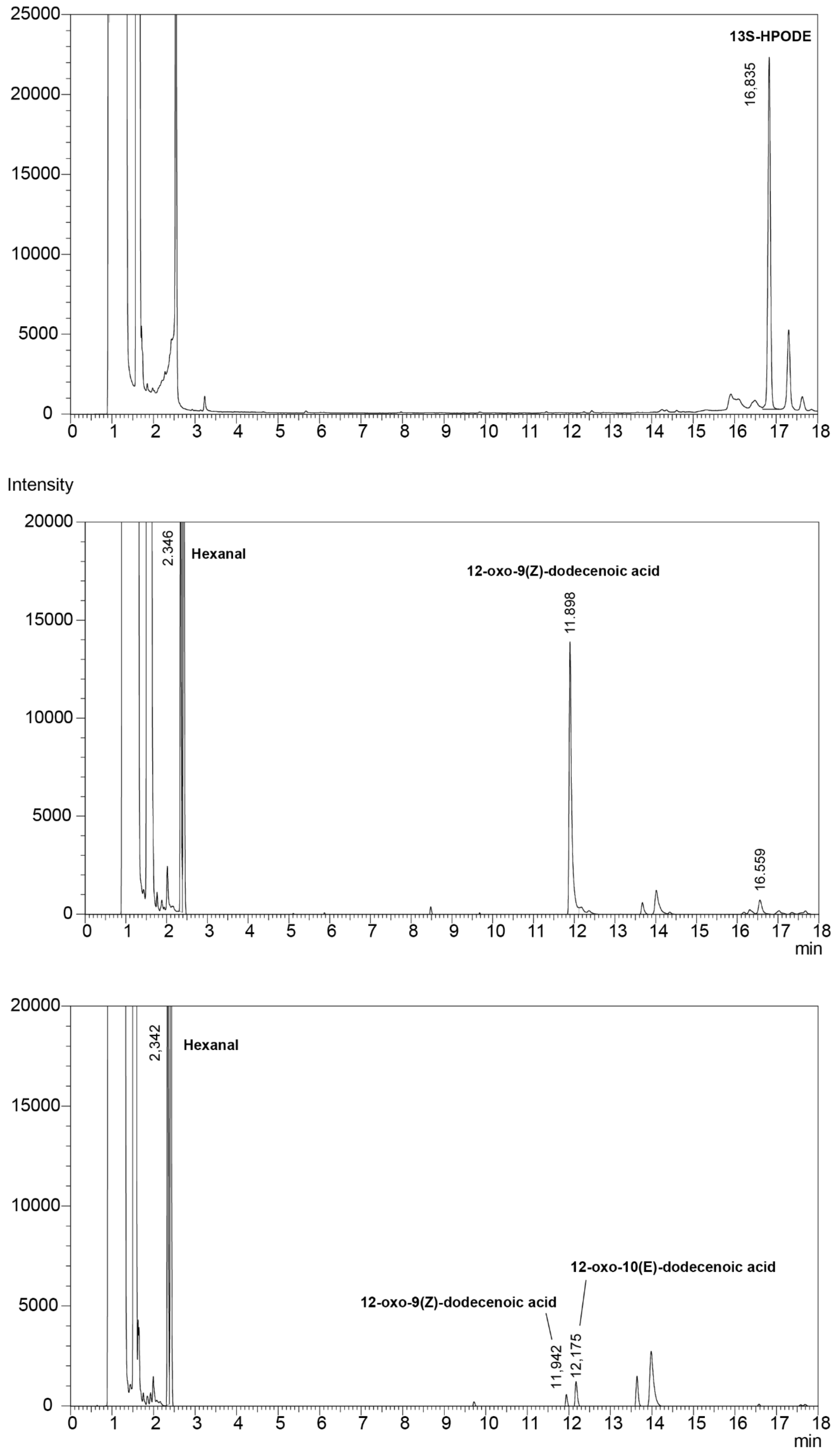


**Fig. S9** GC-FID spectra after sample hydrogenation with sodium borohydride and silylation of **(top)** substrate 13S-HPODE, **(middle)** $HPL_{CP\text{-}N}$ (soluble fraction, 22 °C) reaction for 10 s and **(bottom)** $HPL_{CP\text{-}N}$ reaction for 120 min

**a)**

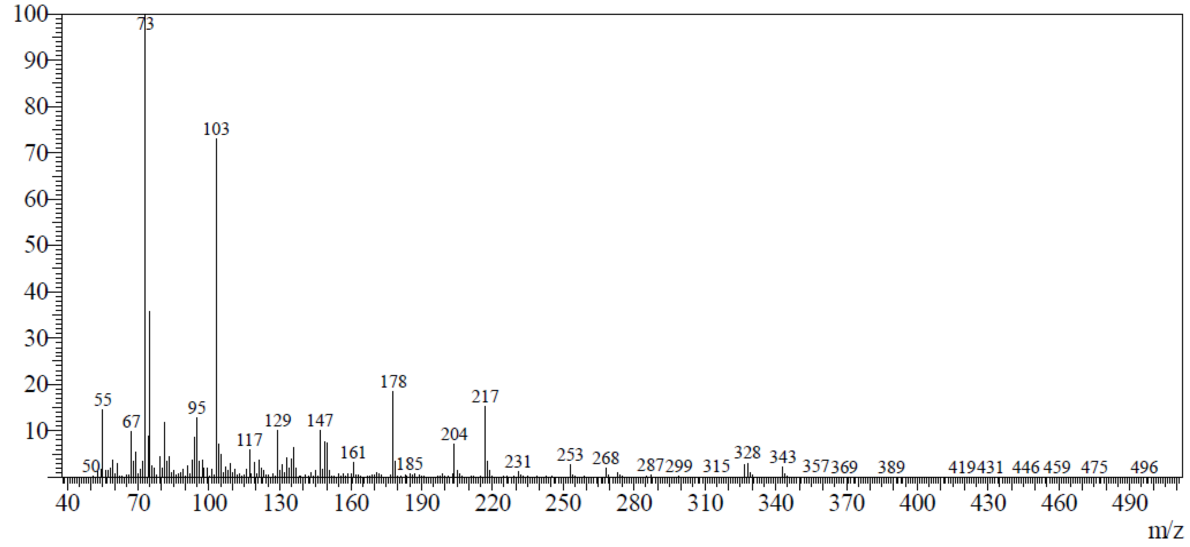

100 90 80 70 60 50 40 30 20 10
73
103
178
217
55 67 95 117 129 147 161 204
50 185 231 253 268 287299 315 328 343 357369 389 419431 446 459 475 496
40 70 100 130 160 190 220 250 280 310 340 370 400 430 460 490
m/z


**b)**

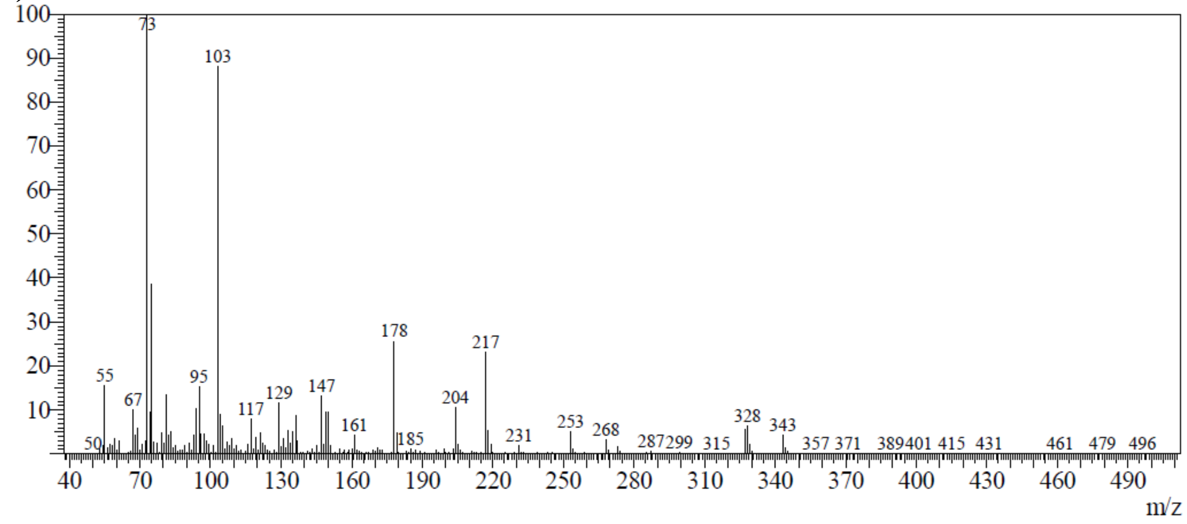

100 90 80 70 60 50 40 30 20 10
73
103
178
217
55 67 95 117 129 147 161 204
50 185 231 253 268 287299 315 328 343 357 371 389401 415 431 461 479 496
40 70 100 130 160 190 220 250 280 310 340 370 400 430 460 490
m/z


**c)**

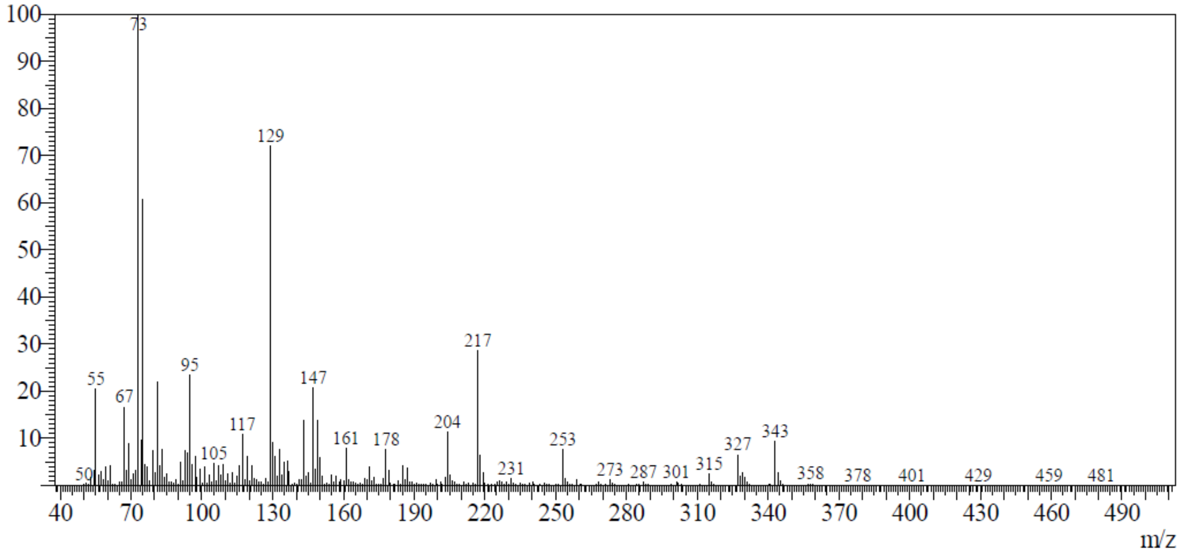

100 90 80 70 60 50 40 30 20 10
73
129
217
55 67 95 117 147 161 178 204
50 105 231 253 273 287 301 315 327 343 358 378 401 429 459 481
40 70 100 130 160 190 220 250 280 310 340 370 400 430 460 490
m/z

**d)**

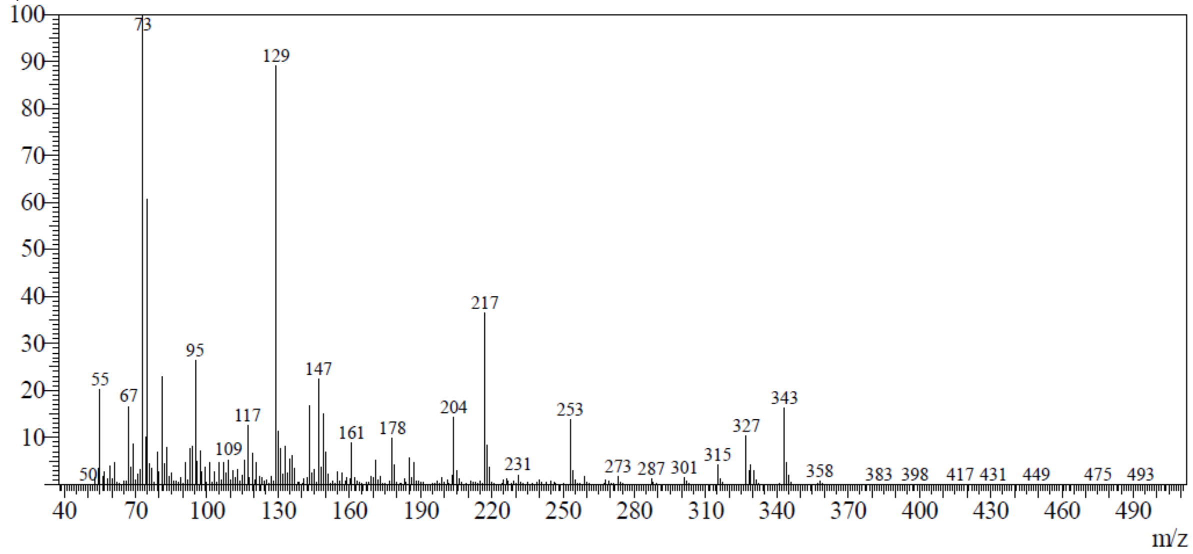


**e)**

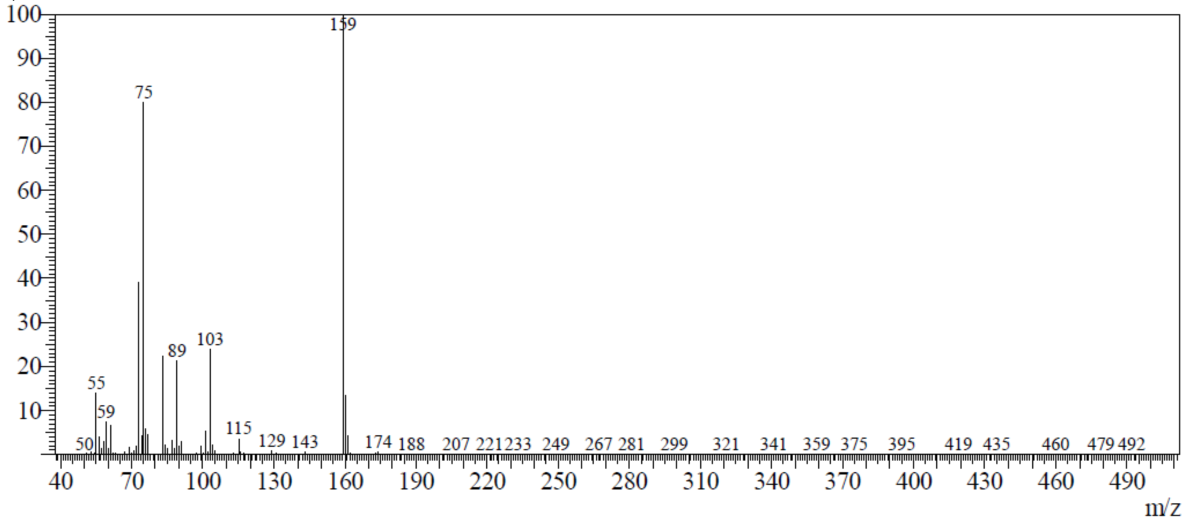


**f)**

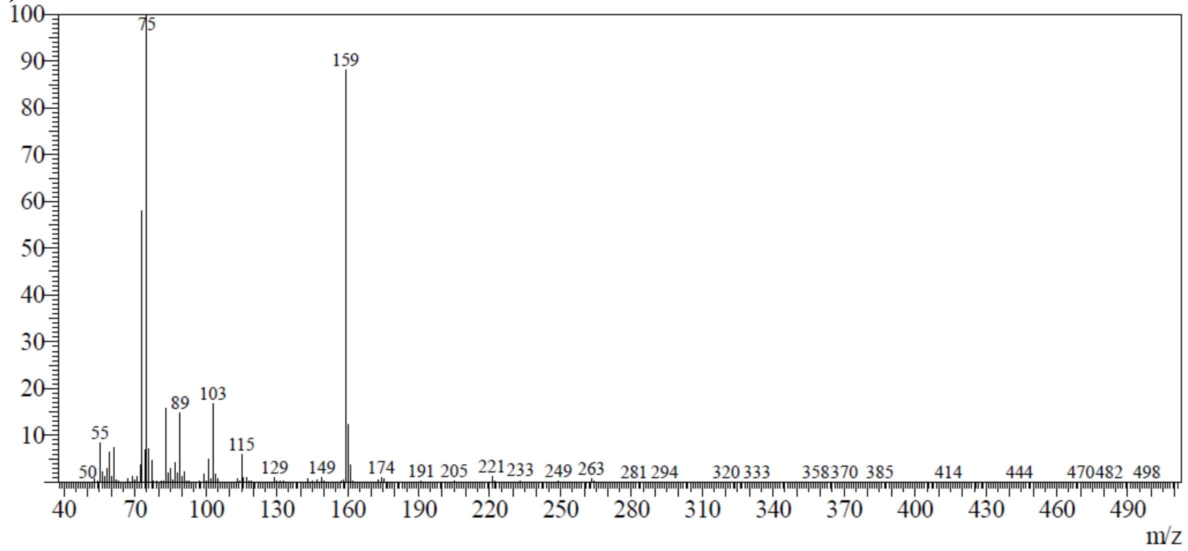


**Fig. S10** Mass spectra of $HPL_{CP\text{-}N}$ reaction products after hydrogenation and silylation **(a, c, e)** in comparison to reference standards from Larodan and Sigma Aldrich **(b, d, e)** with **(a+b)** 12-oxo-9(Z)-dodecenoic acid; **(c+d)** 12-oxo-10(E)-dodecenoic acid (traumatin) and **(e+f)** hexanol
